# Assessing the potential for non-digestible carbohydrates toward mitigating adverse effects of antibiotics on microbiota composition and activity in an *in vitro* colon model of the weaning infant

**DOI:** 10.1093/femsec/fiaf028

**Published:** 2025-03-20

**Authors:** Martha F Endika, David J M Barnett, Emiliana M Olmos, Cajo J F ter Braak, Ilja C W Arts, John Penders, Arjen Nauta, Hans Leemhuis, Koen Venema, Hauke Smidt

**Affiliations:** Laboratory of Microbiology, Wageningen University & Research, 6708 PE Wageningen, The Netherlands; Maastricht Centre for Systems Biology (MaCSBio), Maastricht University, 6229 EN Maastricht, The Netherlands; School for Nutrition and Translational Research in Metabolism, Department of Medical Microbiology, Infection Prevention and Infectious Diseases, Maastricht University Medical Center+, 6229 HX Maastricht, The Netherlands; Laboratory of Microbiology, Wageningen University & Research, 6708 PE Wageningen, The Netherlands; Biometris, Wageningen University & Research, 6708 PB Wageningen, The Netherlands; Maastricht Centre for Systems Biology (MaCSBio), Maastricht University, 6229 EN Maastricht, The Netherlands; School for Nutrition and Translational Research in Metabolism, Department of Medical Microbiology, Infection Prevention and Infectious Diseases, Maastricht University Medical Center+, 6229 HX Maastricht, The Netherlands; FrieslandCampina, 3818 LE Amersfoort, The Netherlands; Avebe Innovation Center, 9747 AA Groningen, The Netherlands; Centre for Healthy Eating & Food Innovation (HEFI), Maastricht University—Campus Venlo, 5928 SZ Venlo, The Netherlands; Laboratory of Microbiology, Wageningen University & Research, 6708 PE Wageningen, The Netherlands

**Keywords:** amoxicillin/clavulanate, azithromycin, 2′-FL, GOS, IMMP, gut bacteria

## Abstract

Environmental factors like diet and antibiotics modulate the gut microbiota in early life. During weaning, gut microbiota progressively diversifies through exposure to non-digestible carbohydrates (NDCs) from diet, while antibiotic perturbations might disrupt this process. Supplementing an infant’s diet with prebiotic NDCs may mitigate the adverse effects of antibiotics on gut microbiota development. This study evaluated the influence of supplementation with 2-fucosyllactose (2′-FL), galacto-oligosaccharides (GOS), or isomalto/malto-polysaccharides containing 87% of α(1→6) linkages (IMMP-87), on the recovery of antibiotic-perturbed microbiota. The TIM-2 *in vitro* colon model inoculated with fecal microbiota of 9-month-old infants was used to simulate the colon of weaning infants exposed to the antibiotics amoxicillin/clavulanate or azithromycin. Both antibiotics induced changes in microbiota composition, with no signs of recovery in azithromycin-treated microbiota within 72 h. Moreover, antibiotic exposure affected microbiota activity, indicated by a low valerate production, and azithromycin treatment was associated with increased succinate production. The IMMP-87 supplementation promoted the compositional recovery of amoxicillin/clavulanate-perturbed microbiota, associated with the recovery of *Ruminococcus, Ruminococcus gauvreauii* group, and *Holdemanella*. NDC supplementation did not influence compositional recovery of azithromycin-treated microbiota. Irrespective of antibiotic exposure, supplementation with 2′-FL, GOS, or IMMP-87 enhanced microbiota activity by increasing short-chain fatty acids production (acetate, propionate, and butyrate).

## Teaser

Supplementing infant diets with specific prebiotics may support gut microbiota recovery after certain antibiotics, promoting microbiota activity, and short-chain fatty acid production during early development.

## Introduction

The microbial communities in the infant gut develop under strong selective pressures, among others introduced by diet-derived nutrients. In particular, the gut microbiota of infants in the weaning period is exposed to solid foods, which mostly contain complex carbohydrates, including pectin from fruits and starches from infant cereal. Significant amounts of starch are likely to enter the infant colon during weaning, due to the lack of chewing and immature pancreatic amylase (Edwards and Parrett 2003). The excretion of starch in feces has been observed in children up to three years of age (Parret et al. 2000). Moreover, the milk intake during the weaning period exposes the gut environment to non-digestible carbohydrates (NDCs), including prebiotics, such as human milk oligosaccharides from breast milk or galacto-oligosaccharides (GOS) from GOS-supplemented infant formula (Gibson et al. 2017).

When a child receives orally administered antibiotics, a fraction of the antibiotic may enter the gut and perturb gut microbiota development. Penicillins (in particular amoxicillin alone or in combination with clavulanate, a β-lactamase inhibitor) and macrolides (e.g. clarithromycin or azithromycin) are among the most commonly prescribed antibiotics in children (Clavenna and Bonati 2011). These antibiotics are used to treat children older than six months for common infections, such as acute otitis media (Le Saux et al. 2016). The gut microbiota of infants appears to be more susceptible to perturbation by amoxicillin than the gut microbiota of children older than 2 years (Korpela et al. 2016, Korpela et al. 2017). As for macrolide treatment, previous studies in children have observed that oral azithromycin reduced microbiota alpha diversity and induced a shift in fecal microbiota composition (Doan et al. 2017, Parker et al. 2017, Wei et al. 2018).

Several approaches have been proposed to counteract the impact of antibiotics on the gut microbiota, including NDC supplementation in the diet to selectively enrich desired microbes (Fassarella et al. 2021). The supplementation with GOS in a follow-on formula was well tolerated by infants at weaning and showed positive effects on the growth of bifidobacteria (Fanaro et al. 2009). Similar to GOS, the supplementation with 2-fucosyllactose (2′-FL) in an *in vitro* gut model showed positive effects on the composition and activity of fecal microbiota from weaning infants and toddlers (Van den Abbeele et al. 2019, Lindner et al. 2023). Besides these established prebiotics, starch-derived NDCs could be promising as dietary supplements during the weaning period. For example, the supplementation with isomalto/malto-polysaccharides (IMMP) has been shown to stimulate the growth of bifidobacteria and lactobacilli in an *in vitro* study using adult fecal microbiota (Gu et al. 2018). Therefore, we conducted this study to evaluate the influence of different NDCs, namely 2′-FL, GOS, or IMMP, on the gut microbiota of weaning infants in the presence of the common antibiotics amoxicillin/clavulanate or azithromycin. Particularly, microbial composition and activity were compared between unperturbed and perturbed microbiota to evaluate whether specific NDCs could affect microbiota recovery and mitigate any adverse effects of antibiotics.

## Materials and methods

### Antibiotics and NDCs

Amoxicillin/clavulanate (amoxicillin trihydrate: potassium clavulanate, 4:1; Sigma–Aldrich, Saint Louis, MO, USA), a broad-spectrum penicillin antibiotic, and azithromycin dihydrate (TCI Europe N.V, Zwijndrecht, Belgium), a broad-spectrum macrolide antibiotic, were used in this study. NDCs tested in this study included purified Vivinal^®^ GOS and Aequival^®^ 2′-fucosylactose (2′-FL) that were both provided by FrieslandCampina Ingredients (Amersfoort, the Netherlands), and Isomalto/malto-polysaccharides (IMMP-87) that was provided by Avebe (Groningen, the Netherlands). Aequival^®^ 2′-FL powder is a high-purity human milk oligosaccharide product (94% 2′-FL on a dry matter basis). Purified Vivinal^®^ GOS contains 91.7% GOS on a dry matter basis. IMMP-87 is a linear glucose polymer with an average molecular weight of 10 kDa, containing 87% α(1→6) linkages and 13% α(1→4) linkages.

### Fecal sample collection and preparation

Fecal samples were collected at nine months postpartum from six vaginally delivered full-term infants, who were not exposed to antibiotics and participated in the Baby Carbs study (Endika et al. 2024). All six infants received both milk and solid food in their diet at this age. The type of solid food consumed by the infants included breads, cheese, baby cereals, fruits, vegetables, peanuts, fish, and meat products. Infant fecal samples were collected by participants, and kept refrigerated under anoxic conditions for a maximum of 72 h, after which the fecal samples were processed in the lab as previously described (Endika et al. 2023). Briefly, fecal samples were weighed inside an anaerobic chamber (Bactron300-2, Sheldon Manufacturing, OR, USA; 96% N_2_, 4% H_2_) and diluted in a sterile anoxic solution of pH 6.5 (Tritium Mikrobiologie B.V., Eindhoven, the Netherlands) containing 2.5 g/L KH_2_PO_4_, 4.5 g/L NaCl, 0.005 g/L FeSO_4_.7H_2_O, 0.05 g/L ox bile, 0.04 g/L cysteine-HCl, and glycerol (final concentration of 10%, v/v). The fecal slurries (25% w/v) were mixed using a vortex, filtered through a sterile sieve, and then aliquoted into sterile 10 ml vials (La-Pha-Pack, Langerwehe, Germany). The vials were sealed with a sterile butyl rubber stopper with a crimp cap. The sealed fecal slurries were taken out from the anaerobic chamber and immediately snap-frozen in liquid nitrogen prior to storage at −80°C.

### Operation of the *in vitro* model of the colon (TIM-2)

**Figure 1.**
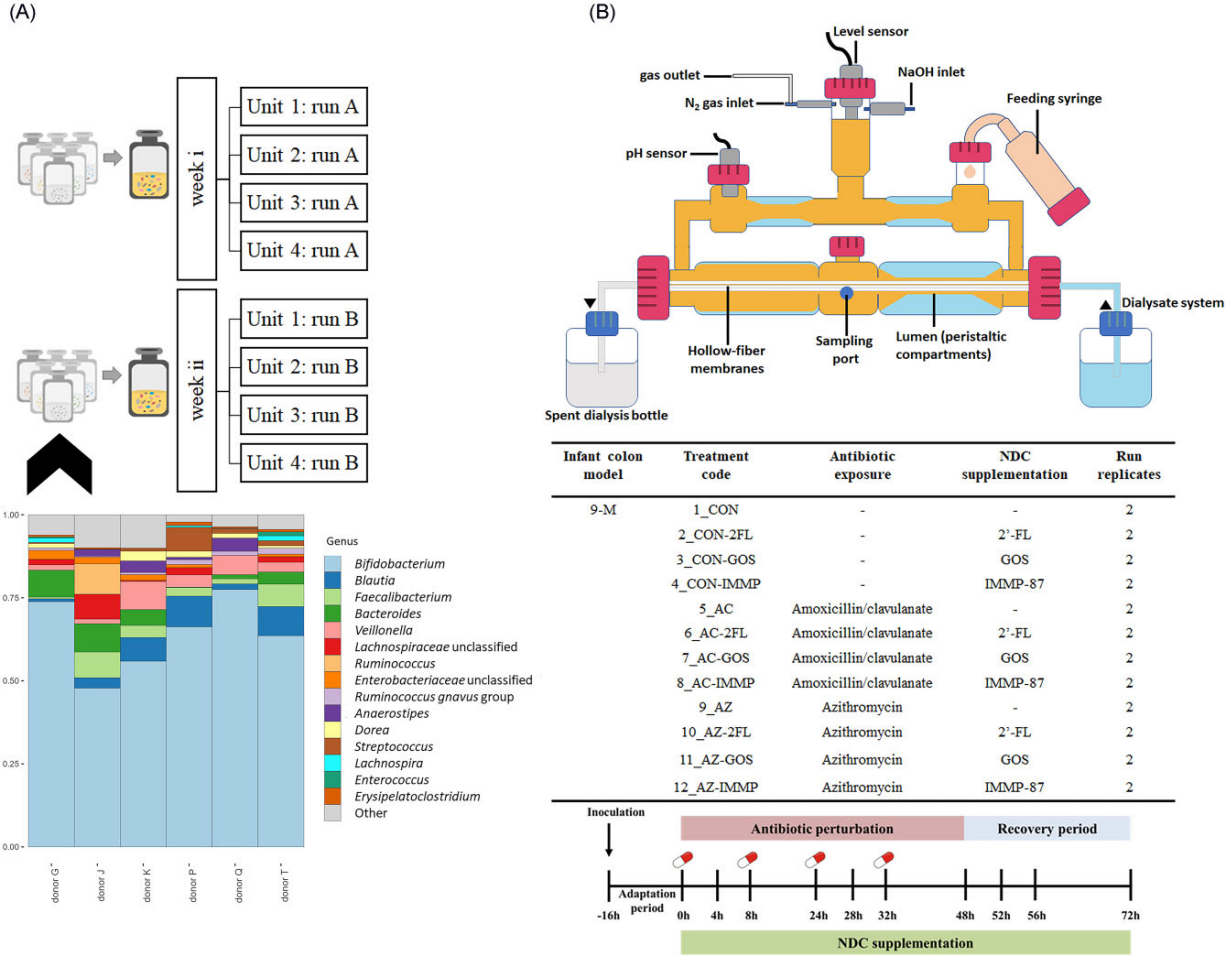
Microbiota composition of individual fecal samples used in pooled inocula (a) and schematic overview of the experimental setup and sampling scheme of the *in vitro* TIM-2 colon model (b). Top 15 genera are shown and other genera are grouped as “Other”. The different donor codes correspond to the six different infant donors whose fecal samples were used in this study. Lumen compartment samples were taken from the sampling port and the dialysis liquid samples were collected from the spent dialysis bottle. A control run without antibiotic and without NDC supplementation was also included as a reference. Each treatment was run in duplicate (run A and B). Four units of TIM-2 were run in parallel.

Frozen infant fecal slurries were thawed in a water bath at 37°C for 30 min and were opened inside an anaerobic chamber (80% N_2_, 10% CO_2_, 10% H_2_). In order to achieve a sufficient volume of fecal material to inoculate each TIM-2 run with a standardized starting microbiota, the fecal slurries from six healthy 9-month-old infants were pooled. The infant fecal slurries were selected for pooling based on their similarity in microbiota composition, as ascertained through 16S ribosomal RNA (rRNA) gene amplicon sequencing (see below for details). The infant fecal slurries from the infants were characterized by the presence of *Faecalibacterium* and *Blautia*, which are typically associated with weaning microbiota. The microbiota composition of individual fecal slurries is provided in Fig. 1.

The lumen compartment of TIM-2 was filled with dialysis liquid (pH 6.0), and the TIM-2 unit was flushed with nitrogen as previously described (Endika et al. 2023). Each TIM-2 unit was inoculated with pooled infant fecal slurry (final concentration of 2.5% w/v) and the total volume was maintained at 120 ml. The temperature was maintained at 37°C, and the pH was controlled between 6.0 and 6.2 by continuous addition of 2 M NaOH.

The simulated ileal efflux medium for weaning infants (i-SIEM W), a concentrated feeding medium modified from De Boever et al. (De Boever et al. 2001), was used to simulate the compounds reaching the colon of infants in the weaning period. The i-SIEM W consisted of the following components (per l): 24.0 g lactose (Merck, Darmstadt, Germany), 6 g tryptone (Oxoid, Basingstoke, UK), 6 g lactalbumin hydrolysate (Merck), 4.5 g pectin from apple (Merck), 6 g starch from rice (Merck), 0.8 g ox bile (Merck), 15 g porcine gastric mucin (partially purified type III; Sigma–Aldrich), 0.6 g urea (Thermo Fisher Scientific, Waltham, MA, USA), 0.4 g cysteine HCl (Merck), 10 ml antifoam B emulsion (Sigma–Aldrich), salt solution (Tritium Mikrobiologie B.V.) containing 4.5 g, NaCl, 2.5 g K_2_HPO_4_.3H_2_O, 0.45 g CaCl_2_.2H_2_O, 0.005 FeSO_4_.7H_2_O, 0.01 g hemin, and 1 ml vitamin solution as mentioned above. Dry heat sterilization was used for rice starch powder for 3 h at 150°C. Other components were autoclaved, except urea and vitamins, which were filter sterilized using sterile 0.2 µm membrane syringe filters (Advance Microdevices, Ambala Cantt, India). The concentrated i-SIEM W was added in the TIM-2 model at a rate of 2.5 ml/h.

The fecal microbiota was allowed to adapt to the gut model for 16 h without the addition of antibiotics and NDCs. After the adaptation period (defined as time point 0), i-SIEM W supplemented with or without NDC was administered over a 72 h period. The NDCs, either 2′-FL, GOS or IMMP-87, were supplemented in the feeding medium at a concentration of 30 g/l, which corresponds to a supplementation of 1.8 g/day.

Figure 1 shows the experimental setup and sampling scheme of the TIM-2 experiment. A pulse of antibiotic solution was administered twice a day at time points 0, 8, 24, and 32 h with a final concentration in the model of 25 μg/ml for amoxicillin/clavulanate or 38 μg/ml for azithromycin. Antibiotic doses of 200 mg of amoxicillin/clavulanate (in two divided doses) and 80 mg of azithromycin per day are the standard pediatric dosage for infants of this age based on an average weight of 8 kg (Dunne et al. 2003, White et al. 2004). The final concentration of antibiotic in the colon model was estimated based on the assumption that 85% of the administered amoxicillin/clavulanate (Le Blay et al. 2009) and 37% of the administered azithromycin (Singlas 1995) was absorbed before reaching the colon. Samples were taken from the lumen compartment, for microbiota and metabolite analysis, and from the dialysate collection bottle, for metabolite analysis, at time points −16, 0, 4, 8, 24, 28, 32, 48, 52, 56, and 72 h. The samples at time points 0, 8, 24, and 32 h were collected just prior to antibiotic addition. The lumen compartment samples were centrifuged for 5 min at 21 000 g to separate the liquid fraction from the microbial biomass. All samples were snap-frozen in liquid nitrogen and stored at −80°C until analysis.

### DNA extraction, quantification of total bacteria, and microbiota composition analysis

Total DNA was extracted from the microbial pellet by a repeated bead beating method (Salonen et al. 2010), followed by an automated purification step using the Maxwell^®^ 16 Instrument (Promega, The Netherlands). Total bacterial 16S rRNA gene copies were quantified by quantitative PCR (qPCR) using a CFX384 Touch^TM^ Real-Time PCR Detection System (Bio-Rad, Hercules, CA, USA), as described previously (Endika et al. 2023), and using BACT1369F/PROK1492R as primer-pair (Suzuki et al. 2000).

Microbial composition was assessed based on the V4 region of the 16S rRNA gene that was amplified from the total DNA in duplicate using barcoded 515F (Parada et al. 2016: 1403–14) and 806R (Apprill et al. 2015) primers, as described previously (Endika et al. 2023). No-template controls were included for each PCR run, not resulting in PCR products when visualized on agarose gels. Subsequently, an equimolar mix of purified PCR products was prepared for each library and sent for sequencing to Novogene (Novogene, Cambridge, UK). Two mock communities of known composition and one no-template control were included for each library. The raw sequence data were processed using NG-Tax 2.0 with default settings (Poncheewin et al. 2020: 1366). Taxonomic assignment of each amplicon sequence variant (ASV) was performed based on SILVA database version 138.1 (Quast et al. 2012).

### Metabolite analysis

A Carrez clarification step was performed on both liquid fractions of samples collected from the lumen compartment and samples collected from the spent dialysis bottle to remove protein from the samples, based on the method described by Selak et al. (2016). The concentrations of organic acids, including succinate, short-chain fatty acids (SCFAs; acetate, propionate, butyrate, and valerate), and branched-chain fatty acids (BCFAs; iso-butyrate and iso-valerate) were determined by high-performance liquid chromatography (Shimadzu LC-2030C Plus, Shimadzu Europa GmbH, Duisburg, Germany), as previously described (Endika et al. 2023). The data was processed using Chromeleon^TM^ CDS software version 7 (Thermo Fisher Scientific).

The ammonia levels in the lumen and dialysate samples were measured in a 96-well microplate (Greiner Bio-One) based on the salicylate-hypochlorite method described by Bower and Holm-Hansen (1980), with modification as described previously (Endika et al. 2023). The absorbance of samples was read at 650 nm using a BioTek Epoch 2 microplate spectrophotometer (Agilent, Santa Clara, CA, USA).

Absolute quantities of each metabolite in the lumen compartment of the TIM-2 model and dialysate bottle were calculated by multiplying the concentration in each sample with the measured volumes. At time point 0 the concentration was artificially set at zero, and the cumulative production of the metabolites after that was calculated.

### Data analysis

Data visualization and analysis were performed in R, version 4.2.0. The absolute abundance of microbial taxa was calculated by multiplying the qPCR count of the total 16S rRNA gene copies with the relative abundance of taxa, following the approach of quantitative microbiome profiling (Jian et al. 2020). Microbiota composition at genus level was visualized using the microViz package version 0.10.8 (Barnett et al. 2021). Taxa that could not be classified at the genus level were renamed to include the lowest classifiable rank, e.g. the genus of *Enterobacteriaceae_*unclassified. To explore associations between antibiotic exposure and the absolute abundances of individual microbial taxa, a simple linear regression model was used to model log2-transformed absolute abundance (after adding a pseudocount of half the minimum abundance to each zero value) of each microbial genus-level taxon at each time point, and the resulting coefficients were visualized using the microViz package. Faith’s phylogenetic diversity was calculated using the picante package version 1.8.2 (Kembel et al. 2010). Non-phylogenetically weighted alpha diversity estimates were calculated using the microViz package, and the exponential of the Shannon index was taken to represent the Shannon effective number of genera. The alpha diversity metrics were visualized using the ggpubr package version 0.4.0 (Kassambara and Kassambara 2020). A two-sample *t*-test was performed to test the differences in alpha diversity between the treatment groups and control group using the rstatix package version 0.7.0 (Kassambara 2021).

To assess beta diversity, principal response curve (PRC) analysis, a special case of redundancy analysis (RDA) (Oksanen et al. 2022), was performed on log2-transformed absolute abundances, allowing the differences between treatments to be interpreted in terms of fold changes. PRC analysis was performed using the rda function in the vegan package version 2.6-4 to test whether NDC supplementation modifies the time-dependent effects of the antibiotic, and vice versa. We fit a separate PRC model for each combination of antibiotic and NDC treatment, using control treatment without antibiotic and NDC addition as the reference condition. In each model, the constraining variables included time, antibiotic treatment, and NDC supplementation, while time was used as a conditioning variable. PRC scores were calculated from each corresponding RDA model, and replicate variability (unconstrained RDA scores) is displayed in the PRC plots using the PRC package version 0.1.1 (ter Braak 2023).

The metabolite data was calculated as cumulative production for metabolites detected in both lumen compartment and spent dialysis liquid, or otherwise presented as detection in lumen compartment only in case of the absence of a given metabolite in the dialysis liquid. To explore associations between antibiotic or NDC treatment and the production of the different metabolites, a simple linear regression model was used to model log2-transformed concentrations of each metabolite per time point, and the resulting coefficients were visualized.

## Results

### Compositional changes in the microbiota exposed to different antibiotics and NDCs

**Figure 2.**
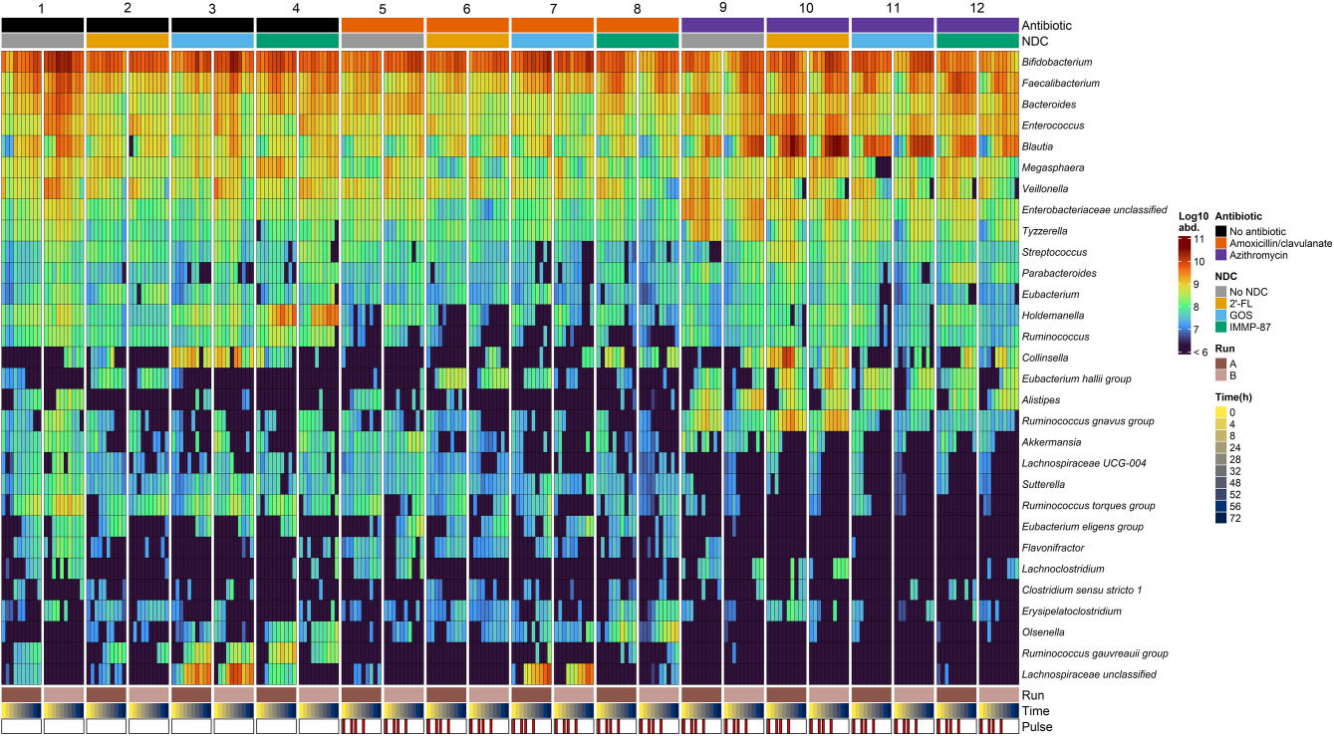
Microbiota composition of each treatment in TIM-2 showing bacterial abundance of the 30 most abundant genera. Samples were grouped by the antibiotic exposure and NDC supplementation. Each treatment was performed in duplicate (runs A and B). Samples were sorted by time, within each treatment group. Antibiotics were added at time points 0, 8, 24, and 32 h (indicated by red annotations under Pulse), immediately after sampling.

In order to assess the potential of different NDCs to mitigate antibiotic-induced perturbations of infant microbiota development at weaning age (i.e. 9 months), we performed TIM-2 experiments for 12 treatments in duplicate. Dynamics in microbiota composition and overall abundance were assessed using 16S rRNA gene targeted amplicon sequencing and qPCR. The total bacterial 16S rRNA gene copies varied over time, and between replicate runs, for each treatment (Supplementary Fig. 1). Therefore, qPCR data were used to correct amplicon-sequence-based compositional data to achieve quantitative microbiota composition profiling based on the absolute abundance of each taxon at genus level at each time point, taking into account the estimated total bacterial load (Fig. 2).

Upon visual inspection of microbiota profiles (Fig. 2), we observed changes in fecal microbiota composition within the colon model, which were dependent on the antibiotic administered and the NDC supplemented in the different treatments. When compared to the microbiota profiles observed in treatments without antibiotics (treatments 1–4 in Fig. 2), we observed changes in the abundance of *Holdemanella, Ruminococcus, R. torques* group and *R. gauvreauii* group in the microbiota of TIM-2 operations exposed to amoxicillin/clavulanate (treatments 5–8). In microbiota of azithromycin-exposed TIM-2 (treatments 9–12), notable changes in the abundances of multiple taxa were seen, including *Blautia, Eubacterium hallii* group, *Alistipes* and *Ruminococcus gnavus* group, among others. It should be noted that azithromycin affected different *Bifidobacterium* and *Blautia* ASVs differently, depending on the specific NDC supplemented in the colon model (Supplementary Fig. 2).

**Figure 3.**
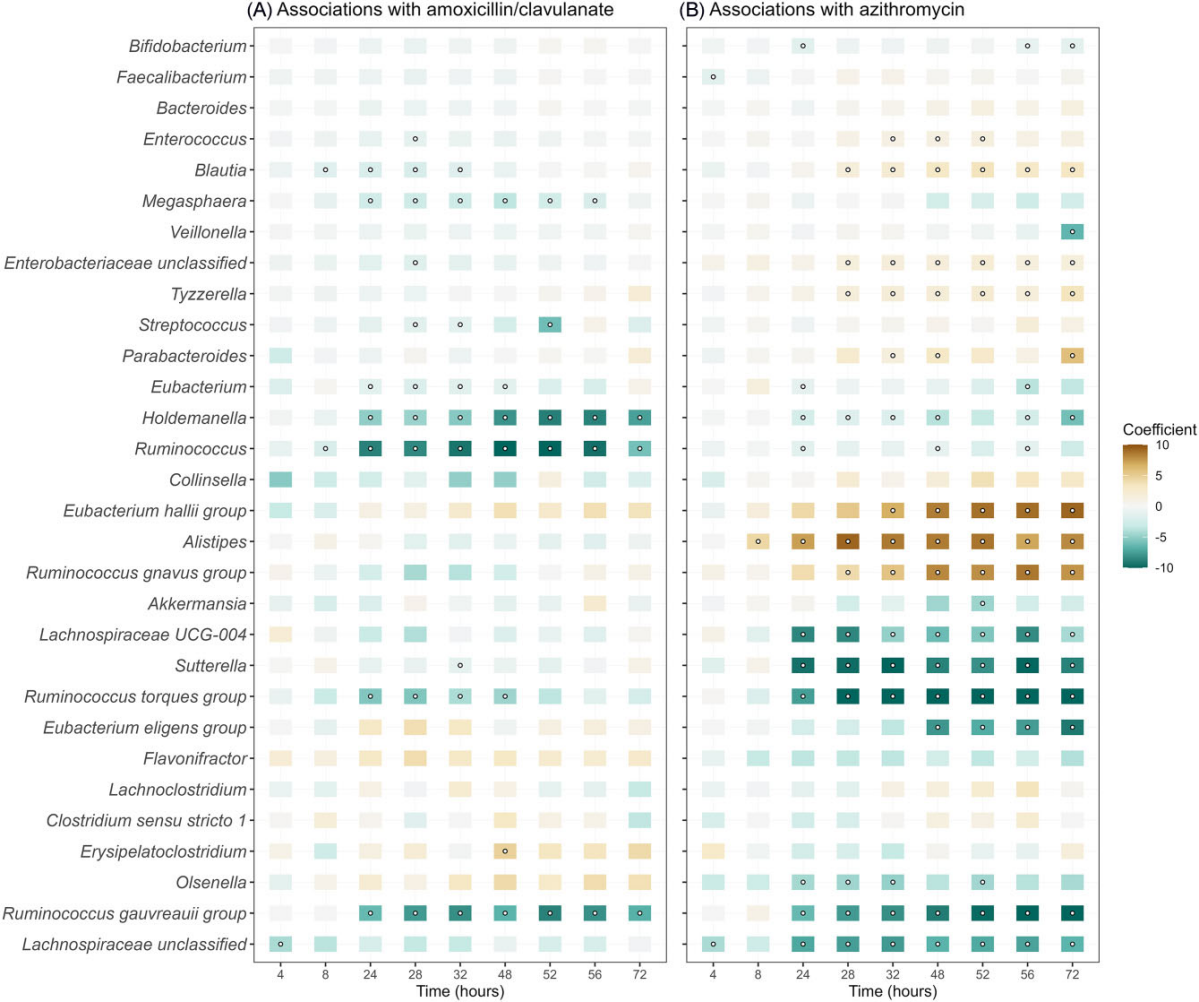
Top 30 most abundant genera and their associations with exposure to amoxicillin/clavulanate (a) and azithromycin (b). Results of simple linear regression models of log2-transformed absolute abundances per time point, per taxon, are visualized in the heatmap. Open circles indicate statistically significant associations (*P* < 0.05). The color of each tile represents the coefficient for the association between each genus and antibiotic.

We further established simple linear regression models (comparing means) to identify significant associations between each antibiotic (as a binary categorical predictor) and bacterial abundances, per time point, per taxon (Fig. 3). Compared to treatments without antibiotics, exposure to amoxicillin/clavulanate was significantly associated with a lower abundance of *Blautia, Megasphaera, Streptococcus, Eubacterium, Holdemanella, Ruminococcus, R. torques group*, and *R. gauvreauii*, at multiple time points, especially from 8 h onward (*P* < 0.05, Fig. 3a). Exposure to azithromycin showed both negative and positive associations with the abundance of several microbial taxa, in most cases at multiple time points, especially from 24 h onward (Fig. 3b). These included associations with increased abundances of *Blautia, Enterococcus*, unclassified genera of *Enterobacteriaceae, Tyzzerella, R. gnavus* group, *E. hallii* group, *Alistipes*, and *Parabacteroides*, as well as with significantly lower abundances of *Bifidobacterium*, unclassified genera of *Lachnospiraceae, Holdemanella, R. torques* group*, Ruminococcus, Eubacterium, R. gauvreauii* group*, Sutterella, Olsenella, E. eligens* group, and *Lachnospiraceae* UCG-004.

### Antibiotic effects on microbial alpha diversity over time

**Figure 4.**
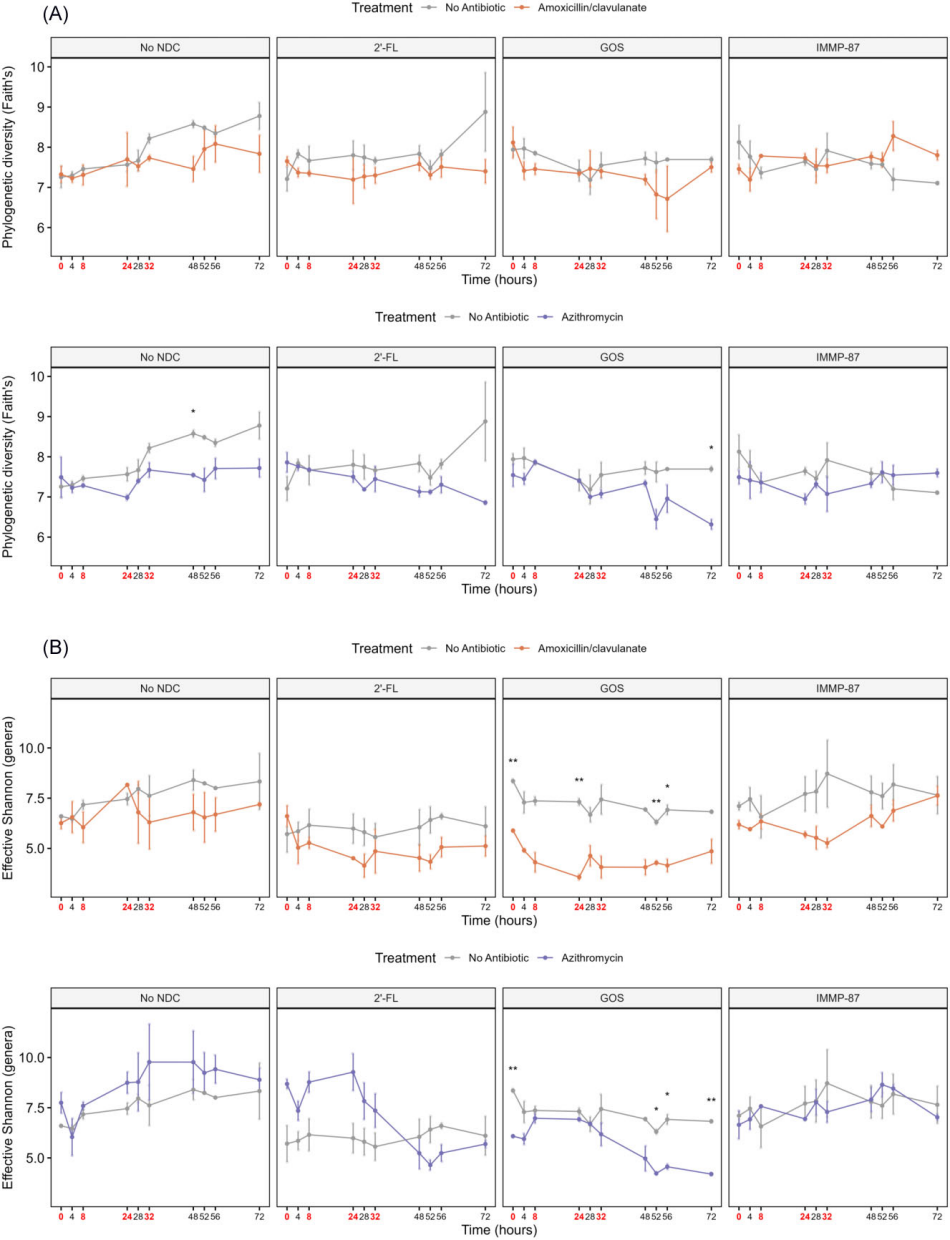
Antibiotic effect on microbial alpha diversity as measured by Faith’s phylogenetic diversity (a) and effective Shannon at genus level (b). Mean values ± SEM are shown. Antibiotics were added at time points 0, 8, 24, and 32 h, immediately after sampling (indicated by red color). Two-sample *t*-tests were used to compare the alpha diversity metrics with and without antibiotic per time point (*P < 0.05 and **P < 0.01 indicates significant differences).

To evaluate the effects of antibiotic exposures on microbial alpha diversity, we measured phylogenetic richness and the exponential of Shannon index (Jost 2019) in each sample (Fig. 4). We did not see a significant effect of amoxicillin/clavulanate on either phylogenetic richness or effective Shannon index of the microbiota in the models with no NDC supplementation. On the other hand, the exposure to azithromycin decreased the phylogenetic richness at time point 48 h but did not affect the effective Shannon index of the microbiota in the TIM-2 models without NDC supplementation.

A similar pattern was seen in the other treatments, except for the colon model supplemented with GOS. The exposure to amoxicillin reduced the phylogenetic richness of the microbiota supplemented with GOS at time point 72 h (Fig. 4a). Moreover, the microbiota in GOS supplemented TIM-2 operations was less diverse (lower effective Shannon) due to amoxicillin/clavulanate exposure at time point 0, 24, 52, and 56 h, and azithromycin exposure at time point 0, 52, 56, and 72 h (Fig. 4b). This might indicate that the effect of the antibiotics tested here on microbial alpha diversity was dependent on the type of NDC supplemented.

### NDC supplementation modified antibiotic effects on microbiota composition

To evaluate the effect of antibiotics and specific NDC supplementation on microbiota composition over time, several PRC analyses were performed. In our initial analyses, described above, we observed that amoxicillin/clavulanate and azithromycin affected bacterial taxa differently. Therefore, we fit PRC models separately for each different combination of antibiotic and NDC to capture the responses of the microbiota to specific antibiotic perturbations and to evaluate the influence of different NDCs per antibiotic, amoxicillin/clavulanate (Supplementary Table 1) or azithromycin (Supplementary Table 2). Each PRC model incorporated data from the colon models with the NDC and the antibiotic combined, the NDC alone, and the antibiotic alone, as well as the condition without antibiotic or NDC, which was used as the reference. For each PRC model, a permutation test indicated that only the first (PRC1) and second PRC axis (PRC2) displayed a significant part of the treatment variance (*P* < 0.05).

In the presence or absence of amoxicillin/clavulanate exposure, we observed that NDC supplementation induced changes in the composition of microbiota, compared to the conditions without NDC supplementation (Supplementary Fig. 3). Overall, the supplementation with NDCs was associated with a decrease in the abundance of mucin-degrading bacteria, including *Akkermansia, Alistipes*, and *Lachnoclostridium*. In turn, the supplementation with 2′-FL was associated with an increased fold change in the abundance of *E. hallii* group, *Erysipelatoclostridium* and *Olsenella*. While the supplementation with GOS was associated with an increase in the abundance of unclassified *Lachnospiraceae* genus-level taxa, *Collinsella*, and *Olsenella*, the supplementation with IMMP-87 was associated with an increased fold change in the abundance of *Olsenella, Collinsella* and *R. gauvreauii* group. Similar patterns were seen in the colon models supplemented with GOS in the presence and absence of azithromycin, while microbiota changes induced by 2′-FL or IMMP-87 were modified in the colon models treated by azithromycin (Supplementary Fig. 4).

**Figure 5.**
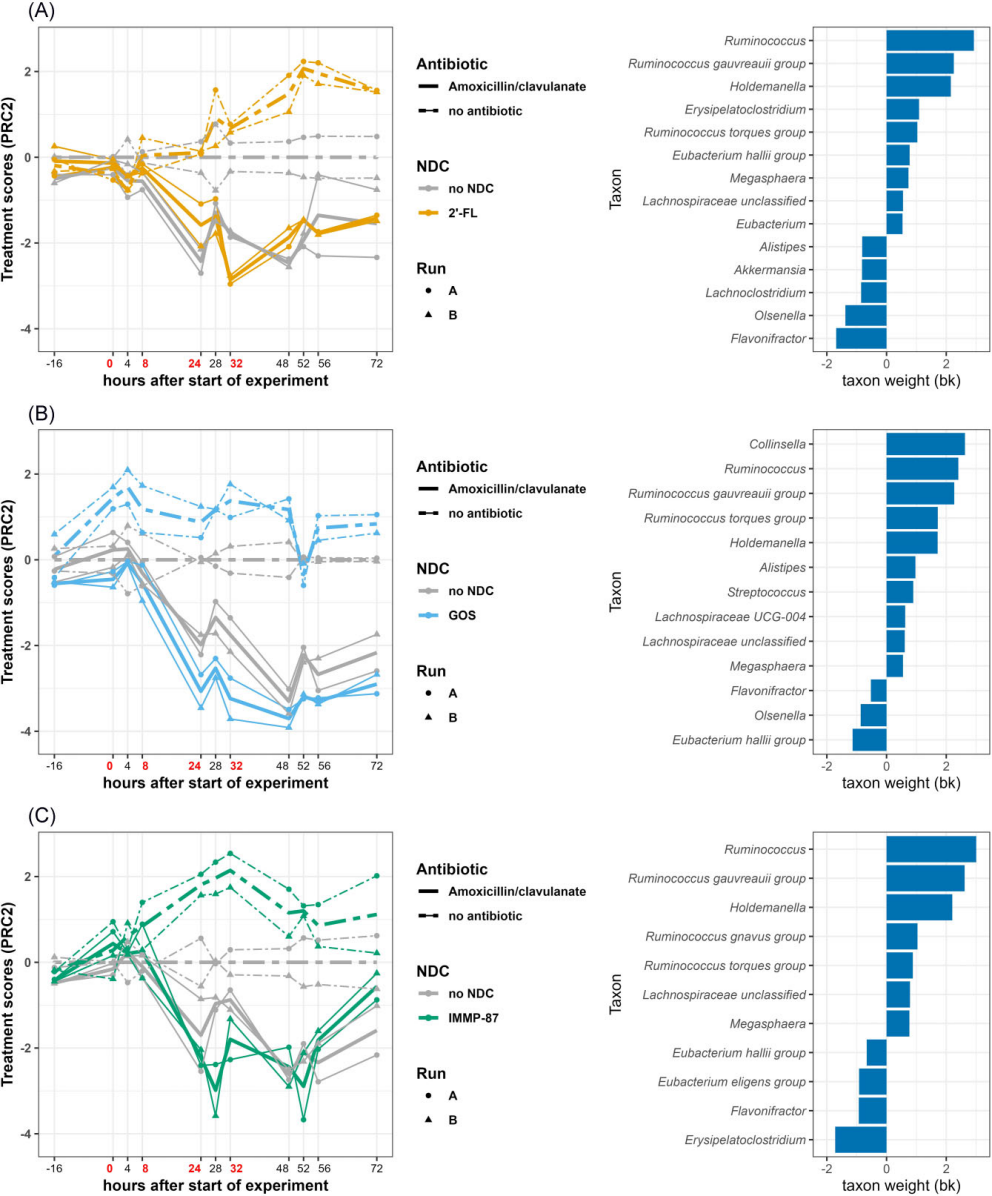
PRC summarizing differences in microbiota composition between antibiotic unexposed and those exposed to amoxicillin/clavulanate, in the colon model supplemented with 2′-FL (a), GOS (b), or IMMP-87 (c). Only the second axis (PRC2) of the PRC models is displayed, accounting for 20% (a), 22% (b), and 22% (c) of the variance in microbiota composition associated with treatment. The analysis was performed on log2-transformed absolute abundances of genera and the PRC scores measure fold-changes. The affinity of a taxon to the PRC2 diagram is shown as taxon weights and taxa with absolute weight above 0.5 are displayed. Antibiotics were added at time points 0, 8, 24, and 32 h, immediately after sampling (indicated by red colored text).

Furthermore, the amoxicillin/clavulanate exposure induced deviation of the microbiota from the unexposed control from time point 8 h onward, displayed in PRC2 (Fig. 5). The deviation induced by amoxicillin/clavulanate was associated with a decrease in the abundance of the genera with positive taxon weight and an increase in the abundance of the taxa with negative taxon weight. Interestingly, signs of recovery were consistently observed in the amoxicillin-perturbed microbiota supplemented with IMMP-87 by time point 72 h. The recovery was associated with an increase in the abundance of *Ruminococcus, R. gauvreauii* group, and *Holdemanella* from time point 52 h onward (Supplementary Fig. 5).

**Figure 6.**
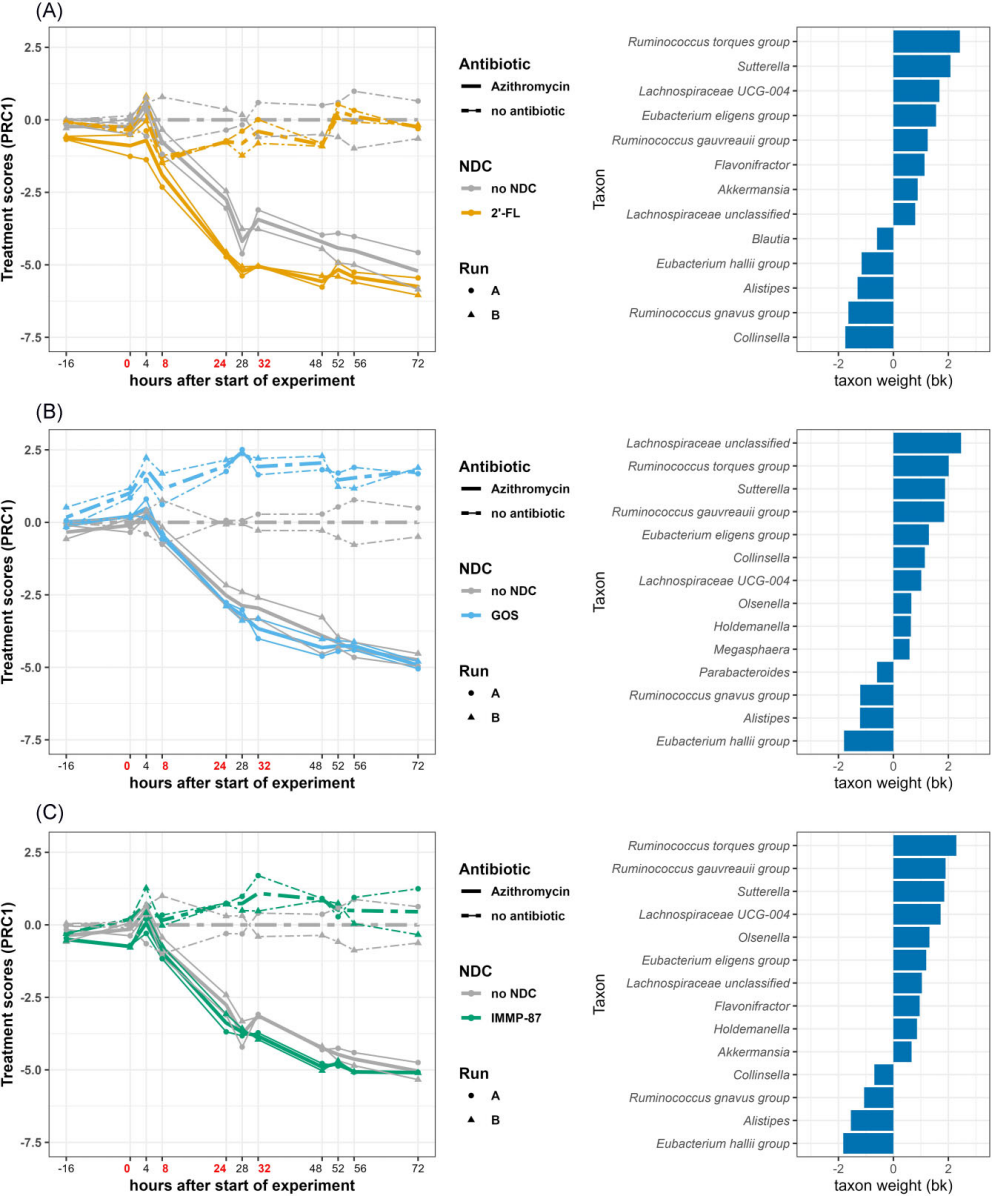
PRC summarizing differences in microbiota composition between antibiotic unexposed treatments and those exposed to azithromycin, in the colon model supplemented with 2′-FL (a), GOS (b), or IMMP-87 (c). Only the first axis (PRC1) of PRC model is displayed, accounting for 19% (a), 21% (b), and 24% (c) of the variance in microbiota composition associated with treatment. The analysis was performed on log2-transformed absolute abundances of genera and the PRC scores measure fold-changes. The affinity of a taxon to the PRC1 diagram is shown as taxon weights and taxa with absolute weight above than 0.5 are displayed. Antibiotics were added at time points 0, 8, 24, and 32 h, immediately after sampling (indicated by red colored text).

In the PRC model on microbiota of azithromycin-exposed and unexposed TIM-2 conditions, the first axis of the PRC (PRC1) displayed a clear deviation in the azithromycin-exposed microbiota from time point 8 h onward, with no indication of recovery (Fig. 6). Azithromycin exposure was associated with decreased abundance of the genera with positive taxon weights, including *R. torques* group, *Sutterella*, and unclassified *Lachnospiraceae* genus-level taxa. Moreover, azithromycin treatment was associated with an increase in the abundance of taxa with negative weights, including *E. hallii* group, *Alistipes*, and *R. gnavus* group.

### Metabolic activity of microbiota in the presence of different antibiotics and NDCs

**Figure 7.**
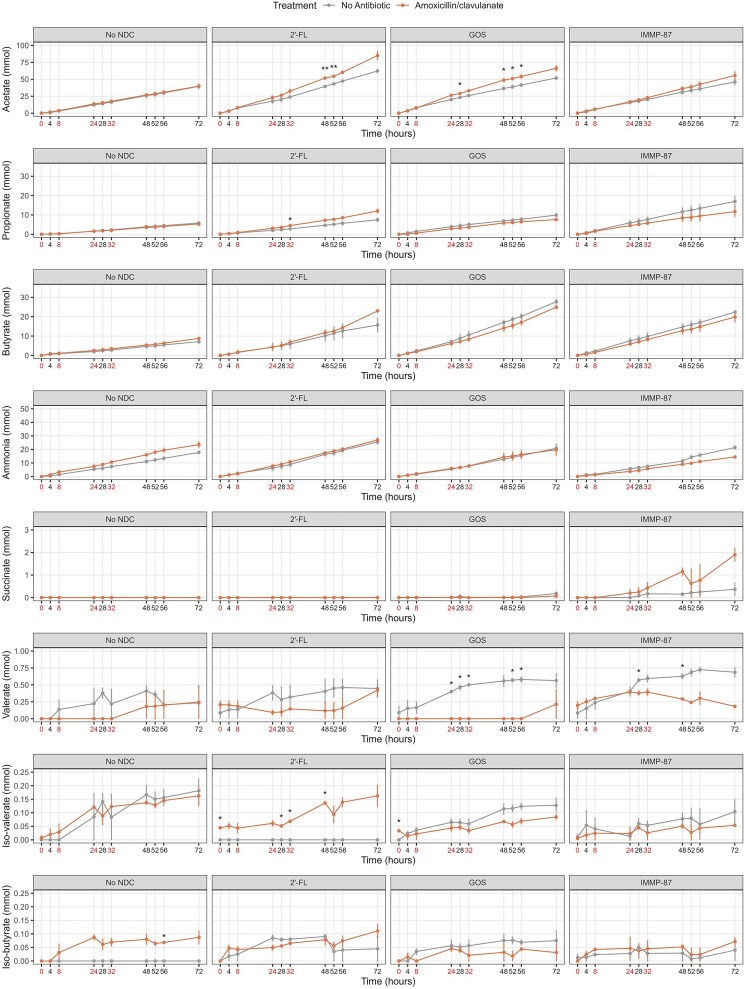
Metabolite production over time in the colon models exposed to amoxicillin/clavulanate and different NDCs. The cumulative production of butyrate, propionate, acetate, and ammonia, along with luminal measurements of succinate, valerate, iso-butyrate, and iso-valerate, are presented. Different scales are used for different metabolites as indicated at the *y*-axes. Antibiotic pulses are indicated by red colored text at time point 0, 8, 24, 32 h. Two-sample *t*-tests were used for comparing the means and significant differences are indicated by *P < 0.05 and **P < 0.01.

To evaluate the effects of antibiotics and NDCs on the production of metabolites, the amounts of succinate, SCFAs, ammonia, and BCFAs during 72 h in TIM-2 operations were determined (Fig. 7). Acetate was the major SCFA produced by the microbiota, followed by butyrate and propionate. Ammonia, BCFAs, and valerate, the metabolites related to protein fermentation, were also produced, albeit in low amounts compared to the SCFAs.

In the absence of antibiotic exposure, the supplementation of 2′-FL was associated with a higher production of acetate and a lower production of iso-valerate from time point 48 to 72 h (Supplementary Fig. 6). Similar to 2′-FL, the GOS supplementation showed significant associations with an increased production of iso-butyrate at multiple time points. We also observed significant associations of GOS or IMMP-87 with a higher production of butyrate and propionate at multiple time points. In addition, increased succinate production was significantly associated with GOS at time point 72 h and IMMP-87 at 48 and 72 h.

**Figure 8.**
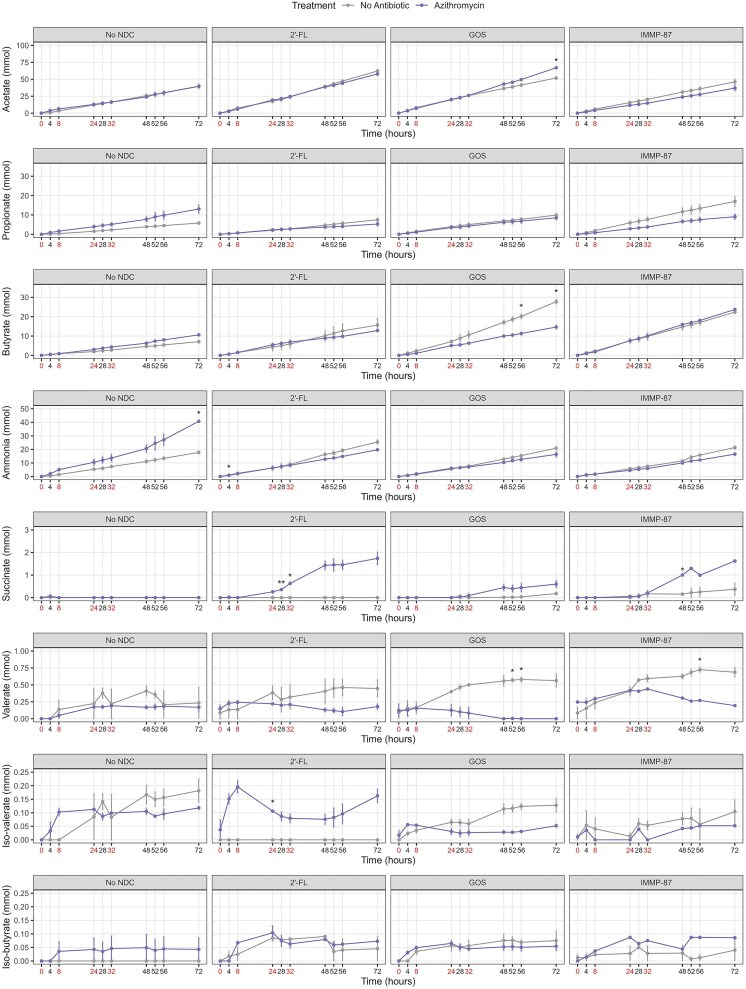
Metabolite production over time in the colon models exposed to azithromycin and different NDCs. The cumulative production of butyrate, propionate, acetate, and ammonia, along with luminal measurements of succinate, valerate, iso-butyrate, and iso-valerate, are presented. Different scales are used for different metabolites as indicated at the *y*-axes. Antibiotic pulses are indicated by red colored text at time point 0, 8, 24, 32 h. Two-sample *t*-tests were used for comparing the means and significant differences are indicated by *P < 0.05 and **P < 0.01.

We observed an increasing trend in iso-butyrate production in the colon model exposed to amoxicillin/clavulanate without NDC supplementation (Fig. 7). On the other hand, an increasing trend in ammonia production was observed in the azithromycin-exposed colon model without NDC supplementation, with a significant difference seen at time point 72 h (Fig. 8). In addition, we observed an increasing trend of succinate production in the azithromycin-exposed colon model supplemented with 2′-FL, with significant differences seen at time point 28 and 32 h. A similar trend in succinate production was also seen in the IMMP-87 supplemented model exposed to azithromycin, with significant differences seen at time point 48 h.

It is worth noting that the amount of acetate produced was higher in the amoxicillin/clavulanate-exposed colon model supplemented with 2′-FL at time point 48 and 56 h or GOS at time point 28, 48, 52, and 56 h. Moreover, a lower valerate production was observed in the antibiotic-exposed colon model supplemented with GOS or IMMP-87 at multiple time points. We also observed an increasing trend in iso-valerate production in the antibiotic-exposed colon model supplemented with 2′-FL, which was not produced in the absence of amoxicillin/clavulanate or azithromycin.

## Discussion

Antibiotic exposure in early life has been shown to exert adverse effects on the composition of the developing gut microbiota, leading to alterations that could have lifelong consequences. Strategies to promote the recovery of perturbed infant gut microbiota following antibiotic exposures include the supplementation with prebiotics. The capacity of disturbed microbiota to return to unperturbed dynamics is defined as resilience (Grimm and Wissel 1997). Therefore, we aimed to investigate the influence of supplementation with 2′-FL, GOS, or IMMP-87, on the resilience of microbiota composition and activity upon exposure to the antibiotics amoxicillin/clavulanate or azithromycin.

The exposure to antibiotics induced changes in the composition and activity of 9-month-old infant feces-derived microbiota in the TIM-2 *in vitro* colon model simulating the weaning period. The effect of NDC supplementation on the recovery of antibiotic-perturbed microbiota was evaluated by assessing the microbiota differences compared to the corresponding unexposed colon models.

We have previously shown *in vitro* that amoxicillin/clavulanate had a strong effect on the feces-derived microbiota from pre-weaning infants (Endika et al. 2023). In line with our findings, both amoxicillin and macrolides, such as azithromycin, were previously found to be strongly associated with compositional changes of the microbiota in a cohort of infants younger than 1 year of age (Korpela et al. 2020). However, in the present study, amoxicillin/clavulanate treatment only affected a few bacterial taxa in the colon model, including different genera within the *Ruminococcaceae*, which contrasted with the broader impact of azithromycin exposure. In a previous study, the lack of gut *Ruminococcaceae* was significantly associated with antibiotic-associated diarrhea in adults receiving amoxicillin/clavulanate (Gu et al. 2022). The survival of other bacteria upon amoxicillin/clavulanate treatment might be explained by the interspecies interactions within the bacterial community. For example, when resistant bacteria produce β-lactamases at levels exceeding the inhibitory effect of clavulanate, other surrounding sensitive bacteria can be protected due to a reduced environmental concentration of β-lactam antibiotics, a phenomenon known as exposure protection (Bottery et al. 2021).

The supplementation with IMMP-87 showed a positive influence on the resilience of microbiota composition upon amoxicillin/clavulanate disturbance by recovering the abundance of ruminococci. It is worth noting that the supplementation with IMMP-87 in the absence of antibiotic led to an elevation in the abundance of *Holdemanella* which includes butyrate-producing *H. biformis*, whereas a previous *in vitro* study showed no effect of IMMP on fecal microbiota composition of 2-weeks-old infants (Logtenberg et al. 2021). Therefore, the supplementation with IMMP-87, a novel type of soluble dietary fiber made from potato starch, might be beneficial during the weaning period, when a more mature microbiota capable of degrading complex polysaccharides is present in the gut.

In contrast to what we observed for amoxicillin/clavulanate, the exposure to azithromycin reduced the abundance of several bacterial genera, similar to an *in vivo* observation among weaning infants in India who received oral azithromycin (Parker et al. 2017). In turn, an increased abundance of *Blautia* was observed following azithromycin treatment, in line with previous human intervention studies in children in an azithromycin-treated group (Doan et al. 2017, Wei et al. 2018). The presence of antibiotic efflux pumps might play a role in the survival of *Blautia* in the presence of azithromycin, as the resistance genes *mtrA* and *macB* were identified in the genome of *B. producta* DSM 2950 (Liu et al. 2021). We also observed that *Alistipes* was positively associated with azithromycin treatment. Interestingly, most *Alistipes* spp. are sensitive to macrolide antibiotics except for *A. obesi*, which was isolated from an obese patient, indicating that variation in the level of resistance to macrolide antibiotics was species/strain-specific (Hugon et al. 2013, Parker et al. 2020). It should be noted that the analyses included in this study, i.e. amplicon sequencing of the V4 region of the 16S rRNA gene, do not provide sufficient resolution to confidently differentiate between different *Alistipes* spp., and thus, future studies addressing this aspect, e.g. through shotgun metagenome analyses, should be considered.

We did not see any influence of NDC supplementation on the recovery of azithromycin-treated microbiota composition, as the antibiotic treatment inhibited the growth of taxa stimulated by specific NDCs or the modulatory effects of a given NDC was associated with the growth of similar taxa enriched by this antibiotic. In particular, the supplementation of 2′-FL further promoted the growth of members of the *E. hallii* group (recently reclassified as *Anaerobutyricum hallii* (Shetty et al. 2018))*¸* one of the bacterial groups that was also found to be associated with azithromycin treatment. *An. hallii*, among the first butyrate producers in early life, was previously identified to have trophic interactions with fucose-utilizing infant *Bifidobacterium* spp. that produce acetate, lactate, and 1,2-propanediol (Schwab et al. 2017). Moreover, *An. hallii* can further metabolize 1,2-propanediol to propanol and propionate (Engels et al. 2016, Shetty et al. 2018). Interestingly, we observed the growth of specific *Blautia* ASVs in the azithromycin-exposed colon model supplemented with 2′-FL. A previous study showed that *Blautia* could use the fucose released from extracellular degradation of 2′-FL (Horigome et al. 2022). In addition, the formate produced during 2′-FL degradation can also be used by *Blautia*, owing to its acetogenic activity (Schwab et al. 2017).

Despite the notable changes in microbiota composition due to azithromycin, the effects of this antibiotic on the metabolic activity of microbiota, particularly SCFA production, were modest, which might be due to the functional redundancy in the microbial communities. Regardless of antibiotic exposure, the supplementation of 2′-FL or GOS led to increased production of acetate and butyrate, in accordance with a previous *in vitro* study in a fecal fermentation model simulating the gut of a toddler between 12 and 18 months old (Lindner et al. 2023). In line with another *in vitro* study using feces-derived microbiota from adults, the addition of IMMP was associated with a significant increase in the levels of SCFAs (propionate and butyrate) and succinate, the latter being a precursor of propionate, which was accompanied by the growth of members of *Bacteroides* that utilize the succinate pathway to form propionate (Gu et al. 2018, An et al. 2021).

The exposure to amoxicillin/clavulanate or azithromycin was associated with a lowered production of valerate, perhaps due to the effect of the antibiotics on *Megasphaera*, as a previous study showed the ability of human gut *Megasphaera* isolates in producing valerate (Shetty et al. 2013). Besides the reduced level of valerate, in the colon model supplemented with NDCs, the exposure to azithromycin was associated with the accumulation of succinate, a direct product of carbohydrate fermentation, which might be linked to the growth of succinate-producing bacteria, including *Alistipes* and *Blautia*, and the absence of succinate-utilizing bacteria such as *Ruminococcus* and *Veillonella*. Succinate increased colonic fluid secretion and the over-accumulation of succinate in the colon can induce diarrhea in weanling piglets (Zhou et al. 2022). On the other hand, NDC supplementation mitigated the azithromycin-induced increase in ammonia production, keeping the ammonia levels produced by the microbiota at their unperturbed level. A previous study observed that high concentration of ammonia impaired the tight junction barrier by increasing oxidative stress in intestinal cells (Yokoo et al. 2021), indicating a negative effect of elevated ammonia production on the host.

In this study, we observed that the type of antibiotic constitutes an important factor in determining the magnitude of deviations from the unperturbed compositional dynamics of microbiota observed in the control TIM-2 conditions. Compared to the compositional changes, the metabolite production of feces-derived microbiota from weaning infants was more resilient to the changes induced by the antibiotics. A challenging task for further research will be the measurement of residual antibiotic or NDC concentrations at specific time points. Another informative line of research would be to carry out the same study using individual feces donations, rather than a pooled fecal inoculum, for evaluating possible individual responses to antibiotic disturbance or NDC supplementation in short- and long-term observations.

## Supplementary Material

fiaf028_Supplemental_File

## Data Availability

The data for this study have been deposited in the European Nucleotide Archive (ENA) at EMBL-EBI under accession number PRJEB64691.
