# Supplementary material for: Assessing the potential for non-digestible carbohydrates toward mitigating adverse effects of antibiotics on microbiota composition and activity in an in vitro colon model of the weaning infant
Source: FEMS Microbiol Ecol. 2025 Mar 20;101(4):fiaf028. doi: 10.1093/femsec/fiaf028 (PMC11963755; doi:10.1093/femsec/fiaf028)
Supplement: fiaf028_Supplemental_File [file fiaf028_supplemental_file.docx]

**Supplementary Information**


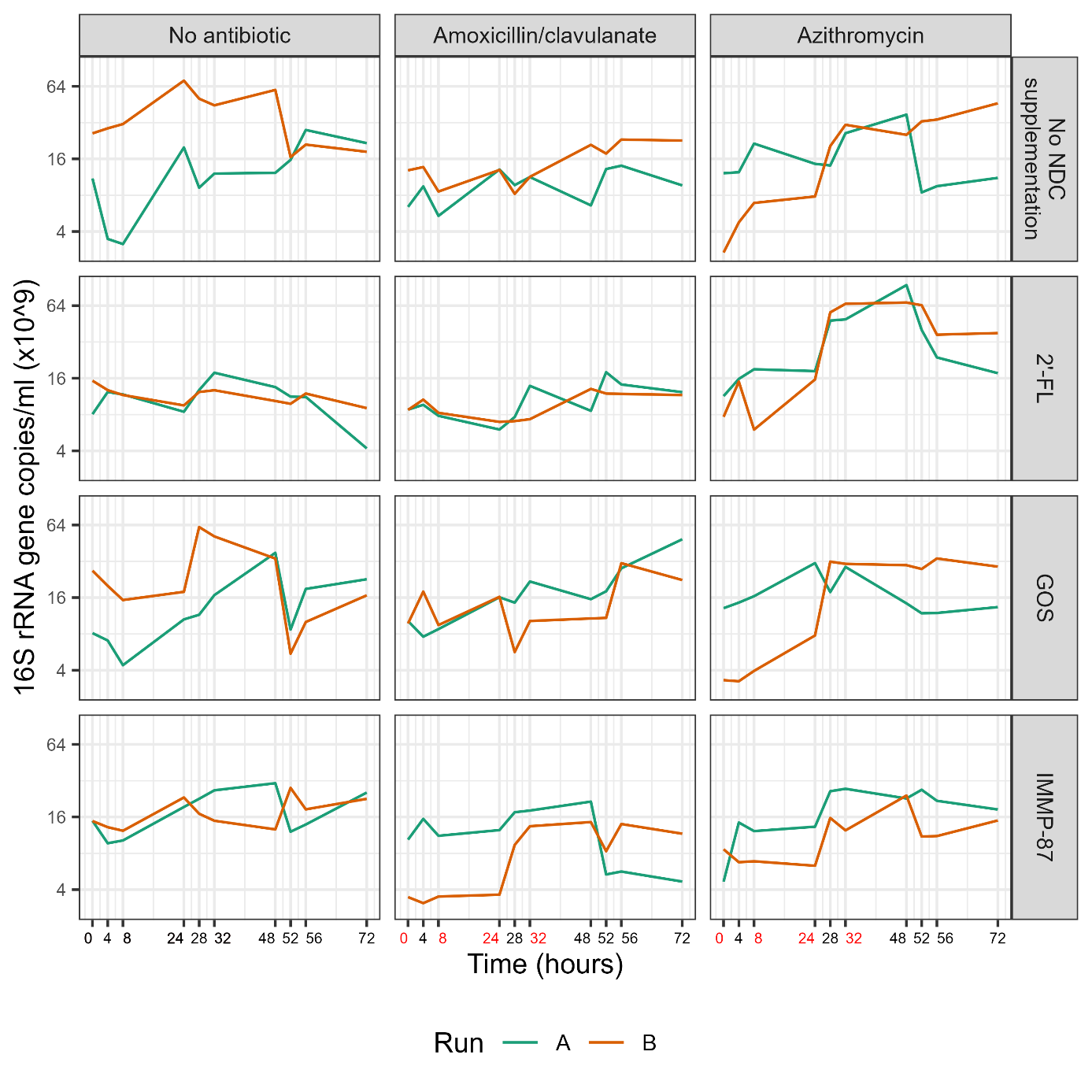
**Supplementary Figure 1.** Total bacterial 16S rRNA gene counts determined by qPCR. Each treatment (with or without antibiotic treatment or NDC supplementation) was run in duplicate (run A and B). Antibiotic pulses are indicated by red colored text at 0, 8, 24, and 32 h.

*
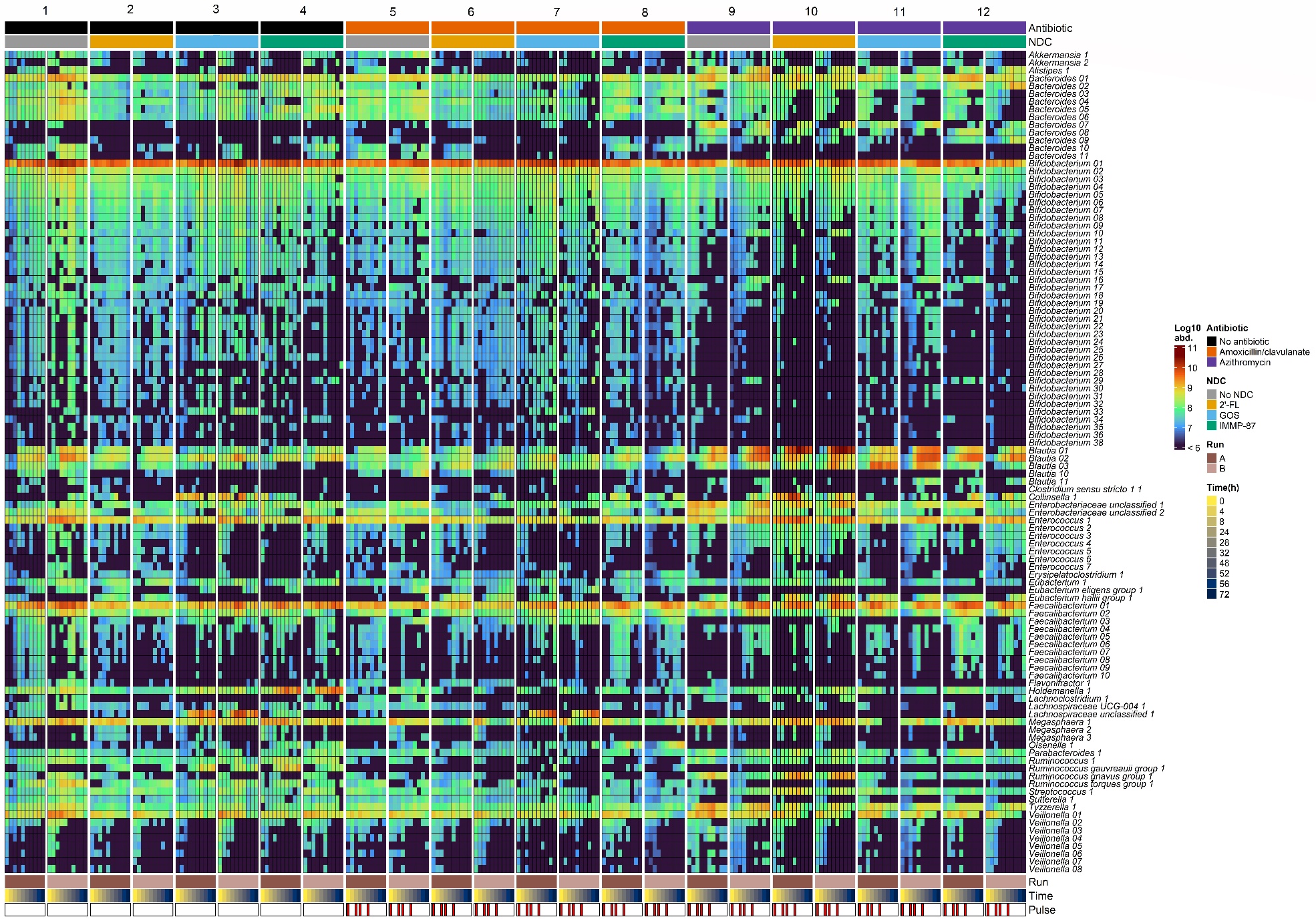
Figure legend on next page*

**Supplementary Figure 2.** Microbiota composition of each treatment in TIM-2 at ASV level. ASVs were sorted in alphabetical order. Samples were grouped by antibiotic exposure and NDC supplementation. Each treatment was performed in duplicate (run A and B). Samples were sorted by operation time for each treatment. Antibiotics were added at time points 0, 8, 24, and 32 h (indicated by red annotations under Pulse), immediately after sampling.


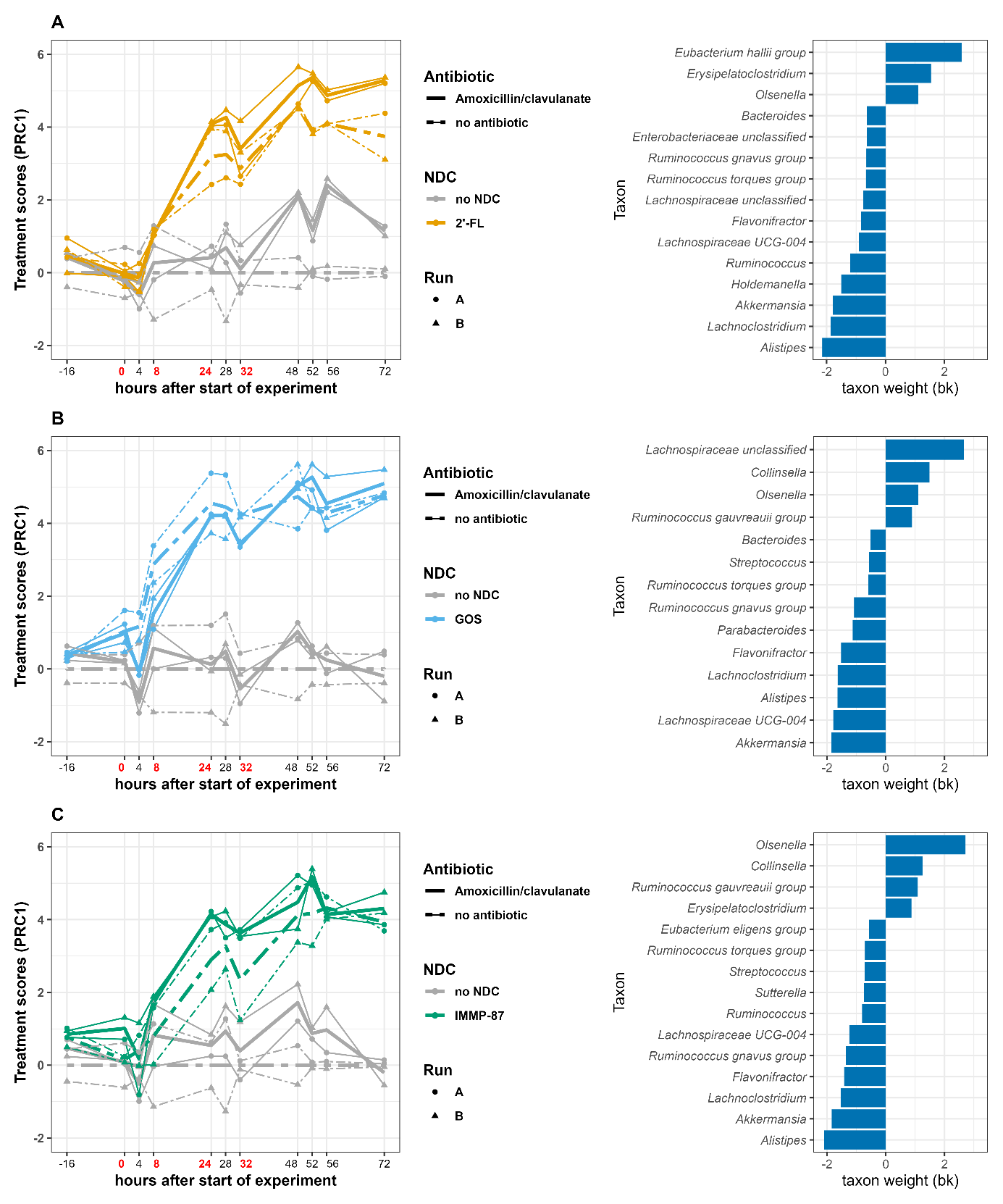


**Supplementary Figure 3.** Principal response curves summarizing differences in microbiota composition between treatments and untreated controls in the TIM-2 colon model supplemented with 2’-FL (A), GOS (B), or IMMP-87 (C), in the presence and absence of amoxicillin/clavulanate. Only the first axis of each PRC model is displayed, accounting for 17% (A), 17% (B), and 19% (C) of the variance in microbiota composition associated with treatment. The analysis was performed on log2-transformed absolute abundances of genera and the PRC scores measure fold-changes. The affinity of a taxon to the PRC1 is shown as taxon weight (bk). Only taxa with bk values above 0.5 or below −0.5 are displayed.


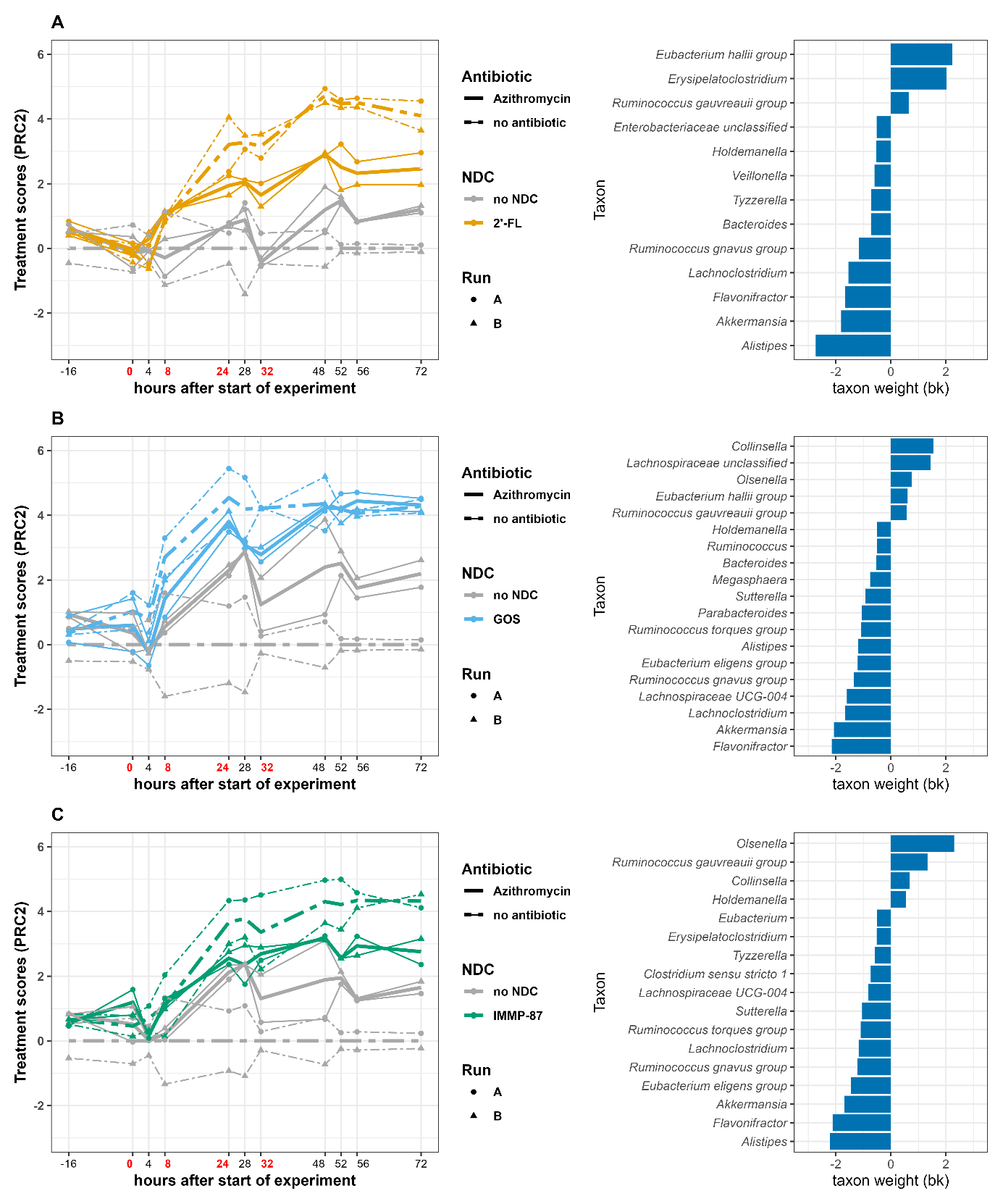


**Supplementary Figure 4.** Principal response curves summarizing differences in microbiota composition between treatments and untreated controls in the colon model supplemented with 2’-FL (A), GOS (B), or IMMP-87 (C), in the presence and absence of azithromycin. Only the second axis of each PRC model is displayed, accounting for 15% (A), 14% (B), and 21% (C) of the variance in microbiota composition associated with treatment. The analysis was performed on log2-transformed absolute abundances of genera and the PRC scores measure fold-changes. The affinity of a taxon to the PRC2 is shown as taxon weight (bk). Only taxa with bk values above 0.5 or below −0.5 are displayed.

*
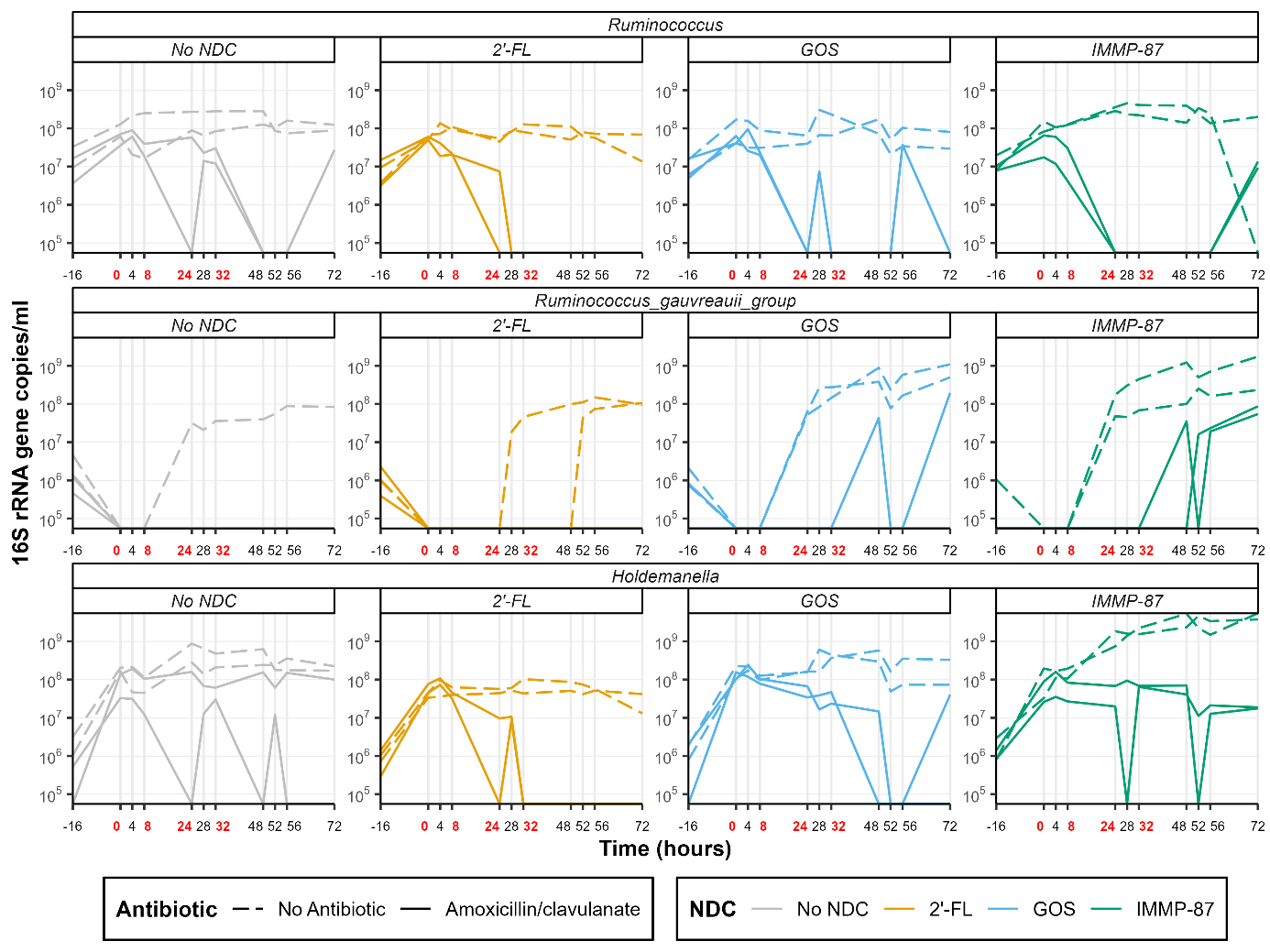
*

**Supplementary Figure 5.** Bacterial abundance calculated based on taxon proportion and the estimated total bacterial 16S rRNA gene counts quantified by qPCR. Top 3 taxa based on absolute bk weight in PRC model of amoxicillin/clavulanate treatment plot are shown. Each treatment was run in duplicate. Antibiotic pulses are indicated by red colored text at 0, 8, 24, and 32 h.


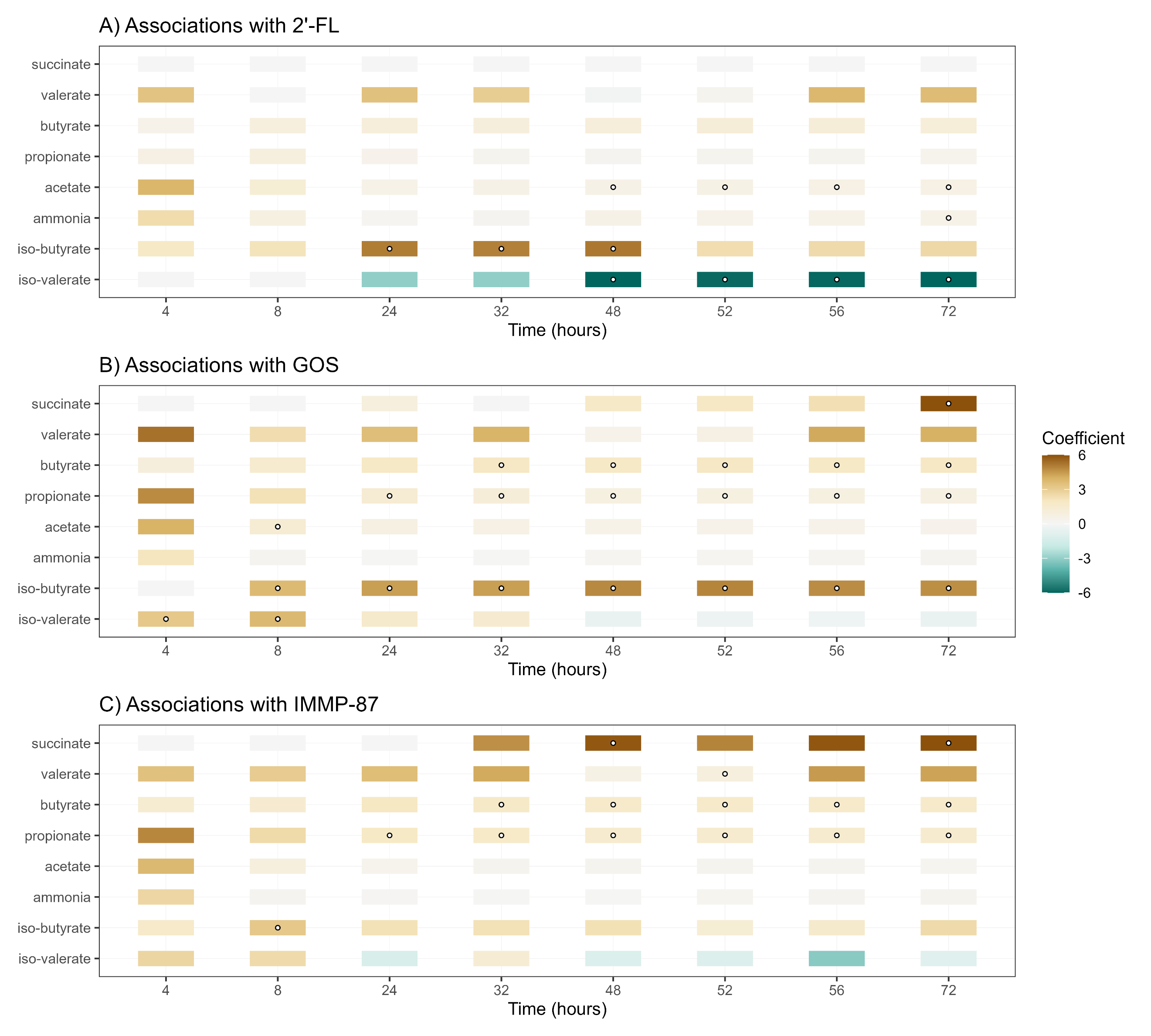


**Supplementary Figure 6.** Metabolites associated with the supplementation of 2’-FL **(A)**, GOS **(B)** and IMMP-87 **(C)**. A summary of the results of simple linear regression models of log2-transformed metabolite amount per time point, per metabolite, are visualized in the heatmap. Open circles indicate statistically significant associations (p < 0.05). The color of each tile represents the coefficient for the association between each metabolite and the exposure to antibiotic.

**Supplementary Table 1.** PRC model of amoxicillin/clavulanate and NDC effect on microbiota

| Amoxicillin/  clavulanate (amox/clav)  model per NDC: | 2’FL  Log abundance ~ Time*Amox/clav*2’FL + Condition(Time) | | GOS  Log abundance ~ Time*Amox/clav*GOS + Condition(Time) | | IMMP-87  Log abundance ~ Time*Amox/clav*IMMP + Condition(Time) | |
| --- | --- | --- | --- | --- | --- | --- |
|  | Inertia | Proportion | Inertia | Proportion | Inertia | Proportion |
| Total | 387.179 | 1.000 | 462.902 | 1.000 | 426.739 | 1.000 |
| Conditional | 126.106 | 0.326 | 135.871 | 0.291 | 131.024 | 0.307 |
| Constrained | 157.662 | 0.407 | 224.693 | 0.482 | 177.707 | 0.416 |
| Unconstrained | 103.411 | 0.267 | 105.338 | 0.226 | 118.007 | 0.276 |

**Supplementary Table 2.** PRC model of azithromycin and NDC effect on microbiota

| Azithromycin (azith) model per NDC: | 2’FL  Log abundance ~ Time*Azith*2’FL + Condition(Time) | | GOS  Log abundance ~ Time*Azith*GOS + Condition(Time) | | IMMP-87  Log abundance ~ Time*Azith*IMMP + Condition(Time) | |
| --- | --- | --- | --- | --- | --- | --- |
|  | Inertia | Proportion | Inertia | Proportion | Inertia | Proportion |
| Total | 446.219 | 1.000 | 475.865 | 1.000 | 450.111 | 1.000 |
| Conditional | 143.801 | 0.322 | 139.860 | 0.294 | 138.314 | 0.307 |
| Constrained | 210.573 | 0.472 | 246.343 | 0.518 | 207.022 | 0.460 |
| Unconstrained | 91.845 | 0.206 | 89.662 | 0.188 | 104.775 | 0.233 |
